# Case Report: Early multidisciplinary rehabilitation and longitudinal functional outcomes in a child with a maternally derived 15q11.2-q13.1 copy-number gain

**DOI:** 10.3389/fped.2026.1943381

**Published:** 2026-09-14

**Authors:** Jingyao Chen, Wangqingyuan Wang, Zijuan Wang, Lihui Liao

**Affiliations:** 1 WCSUH-Tianfu·Sichuan Provincial Children’s Hospital (Sichuan Provincial Children’s Medical Center), Meishan, Sichuan, China; 2 Tianfu College of Southwestern University of Finance and Economics, Chengdu, Sichuan, China; 3 Department of Children’s Rehabilitation, West China Second University Hospital, Sichuan University, Chengdu, Sichuan, China; 4 Ministry of Education, Key Laboratory of Birth Defects and Related Diseases of Women and Children (Sichuan University), Chengdu, Sichuan, China

**Keywords:** case report, developmental delay, Dup15q syndrome, maternally derived 15q11.2-q13.1 copy-number gain, pediatric occupational therapy, rehabilitation

## Abstract

Dup15q syndrome is a rare neurodevelopmental disorder associated with developmental delay, hypotonia or motor impairment, epilepsy or abnormal EEG findings, language impairment, and autism-related social-communication difficulties. This case report describes the clinical phenotype, rehabilitation management, and longitudinal functional trajectory of a male child with a maternally derived 15q11.2-q13.1 copy-number gain. The child presented in early infancy with feeding difficulty, delayed motor development, abnormal sleep EEG findings, delayed white-matter maturation on brain MRI, and later clinically significant autism-related social-communication impairment. Trio whole-exome sequencing with CNV analysis identified an approximately 8.21-Mb *de novo* 15q11.2-q13.1 copy-number gain. MS-MLPA demonstrated four copies across BP1-BP3 and approximately 75% SNRPN methylation, supporting maternal origin. Multidisciplinary rehabilitation began at 5 months and included physical, occupational, speech-language, feeding-related, caregiver-mediated, and later SMO-supported interventions. Serial BSID-II and GMFM-88 assessments were used descriptively, and ADOS-2 characterized autism-related behaviors. Developmental and gross motor scores increased over follow-up, and clinically meaningful milestones including stable head control, independent sitting, crawling, supported standing, and later independent walking were observed. However, because this is a single case without a comparator and follow-up overlapped with early childhood maturation, these changes should not be interpreted as direct evidence of treatment efficacy. This case highlights the value of phenotype-informed rehabilitation planning, developmental surveillance, caregiver involvement, and cautious interpretation of longitudinal outcomes in rare pediatric neurogenetic disorders.

## 1 Introduction

Maternal Dup15q syndrome is a rare chromosomal disorder caused by maternally derived increased dosage of the imprinted 15q11.2-q13.1 Prader-Willi/Angelman syndrome critical region. This increased dosage may result from interstitial duplication or triplication, or from isodicentric chromosome 15 [idic(15)], with severity varying by molecular configuration. The syndrome is phenotypically heterogeneous and is among the more common cytogenetic findings associated with autism spectrum disorder (ASD), with an estimated prevalence of approximately 1 in 30,000-60,000 live births (1, 2).

Clinically, individuals with Dup15q syndrome typically present with a constellation of neurodevelopmental impairments, including global developmental delay, central hypotonia, intellectual disability, language and communication deficits, epileptic seizures, and behavioral characteristics consistent with ASD (3). Early delayed achievement of motor and cognitive milestones are often the first signs noted in infancy. Despite ongoing advances in understanding the genetic and neurobiological underpinnings of Dup15q, current treatment strategies remain largely supportive, lacking specificity and consensus. Management typically involves a combination of pharmacologic seizure control and non-pharmacological therapies such as physiotherapy, occupational therapy, and speech-language intervention (2).

We report a child with maternal Dup15q syndrome supported by a *de novo* maternally derived 15q11.2-q13.1 copy-number gain. The child presented with early feeding difficulty, delayed motor development, abnormal EEG findings, delayed white-matter maturation on MRI, and later autism-related social-communication impairment. By integrating genetic, neuroimaging, neurophysiological, behavioral, and functional information, this report may provide a practical reference for phenotype-informed rehabilitation planning in rare neurodevelopmental disorders.

## 2 Case description

### 2.1 Patient information and initial clinical findings

A male infant was referred to a pediatric rehabilitation service at approximately 5 months of age because of delayed developmental milestones. Baseline BSID-II and GMFM-88 assessments were completed at 4 months and 28 days, immediately before formal multidisciplinary rehabilitation began. He was born by cesarean delivery at 38 weeks and 3 days of gestation, with a birth weight of 2,850 g and length of 48 cm. There was no history of perinatal asphyxia or resuscitation. The parents were healthy and non-consanguineous, and the family history was negative for known neurodevelopmental or seizure disorders.

At the initial examination, the child was alert but showed limited facial expression and reduced social responsiveness. Subtle dysmorphic features included a narrow cranial contour and low-set ears. Neurological assessment revealed abnormal muscle tone, including early upper-limb hypotonia and brisk lower-limb deep tendon reflexes; no pathological reflexes were elicited. Cardiopulmonary and abdominal examinations were unremarkable.

### 2.2 Diagnostic assessment

Hematologic and biochemical testing, including liver and renal function, electrolytes, glucose, lipids, lactate, and ammonia, showed no clinically relevant abnormalities. Blood and urine metabolic screening, serum 25-hydroxyvitamin D, fundus examination, auditory brainstem response testing, abdominal ultrasonography, and cranial ultrasonography were unremarkable.

A 4-hour video EEG at 1 year and 6 months showed occasional low-to-medium amplitude sharp waves over the frontal regions during sleep, more prominently on the left. A caregiver-identified head-shaking event lasting approximately 33 s was captured without ictal epileptiform discharge or electrographic evolution. The EEG was interpreted as borderline abnormal for age. Abundant generalized low-to-medium amplitude fast activity was described during wakefulness and light sleep, but excessive fast beta activity was not explicitly quantified or labeled. This was the only available neurophysiological assessment, and no recurrent clinically confirmed seizures were documented thereafter.

Brain MRI was performed twice during infancy. At 4 months, MRI showed a mildly enlarged cisterna magna, mild focal fullness of the lateral ventricles, mildly widened bilateral frontotemporal extra-axial spaces, and persistent cavum vergae, without definite abnormal parenchymal signal. At 11 months and 2 days, conventional T1-weighted, T2-weighted, and T2-FLAIR MRI showed reduced bilateral cerebral white-matter volume and patchy periventricular white-matter abnormalities near the posterior horns, with low T1WI and high T2WI/T2-FLAIR signals. These findings were interpreted clinically as delayed white-matter maturation.

Trio whole-exome sequencing combined with methylation-specific multiplex ligation-dependent probe amplification of the Prader-Willi/Angelman syndrome region identified an approximately 8.21-Mb copy-number gain at 15q11.2-q13.1 spanning the recurrent BP1-BP3 interval **(**Figure 1). The same change was absent in both parents, supporting a *de novo* event. MS-MLPA showed four copies across BP1-BP3 and approximately 75% SNRPN methylation, supporting maternal origin and maternal Dup15q syndrome. Karyotyping and FISH were not performed, so the structural configuration could not be classified as idic(15), interstitial triplication, or interstitial duplication. A paternally inherited 1q22 duplication was classified as a variant of uncertain significance.

**Figure 1:**
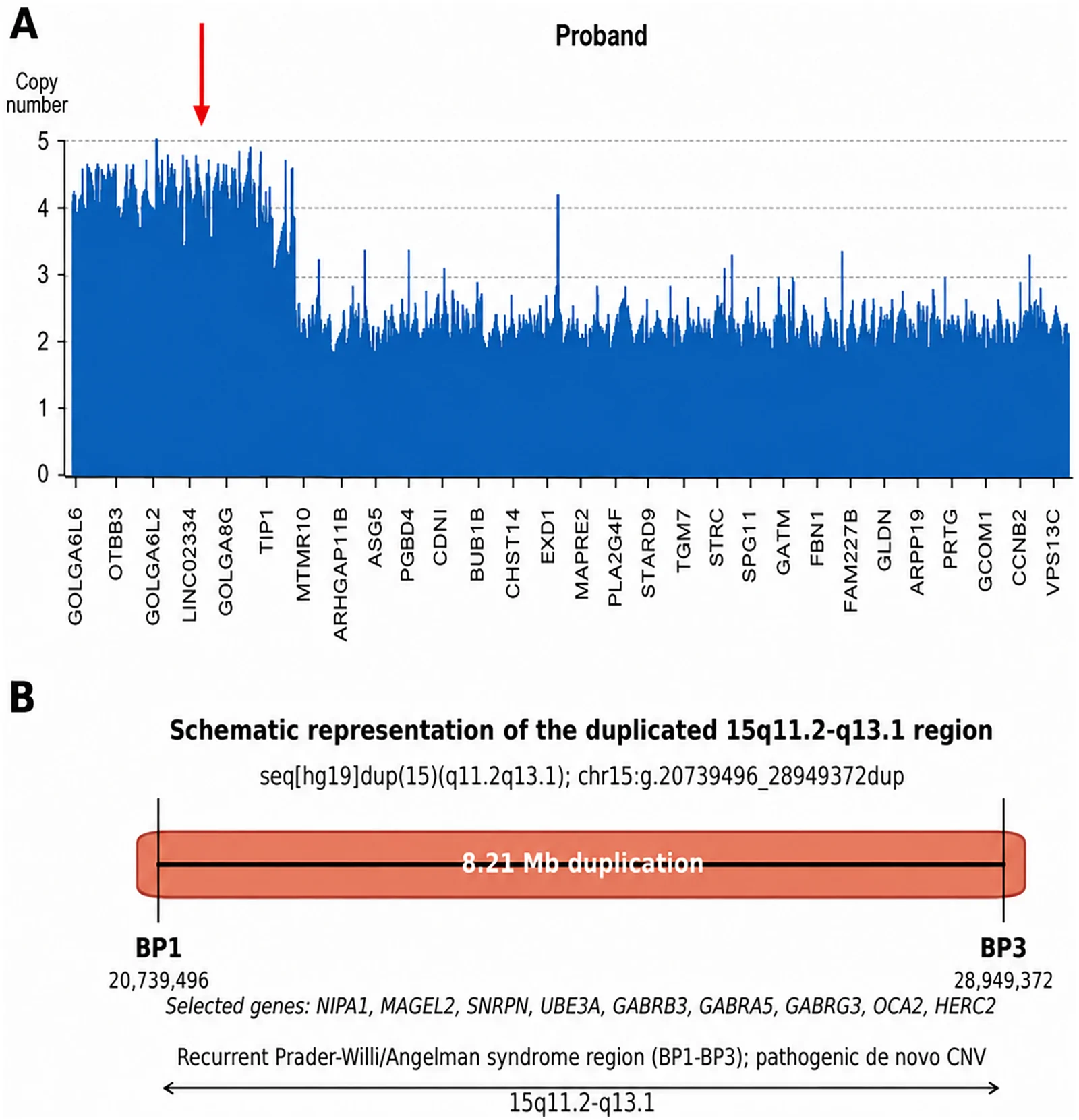
Copy-number analysis of the maternally derived 15q11.2-q13.1 copy-number gain. **(A)** Increased copy number in the proband; the red arrow indicates the involved interval. **(B)** Schematic of the approximately 8.21-Mb pathogenic BP1-BP3 copy-number gain, seq[hg19]dup(15)(q11.2q13.1), chr15:g.20739496_28949372dup. The variant was absent in both parents, supporting a *de novo* origin.

### 2.3 Rehabilitation programs

An individualized, stage-based multidisciplinary rehabilitation program was initiated at 5 months of age in response to the child's early global developmental delay. The program was organized according to functional developmental level rather than chronological age alone. It included physical therapy, occupational therapy, speech-language intervention, feeding-related guidance when clinically indicated, caregiver-mediated home training, and orthotic management when standing and gait-preparation goals became relevant (4). The main intervention components, treatment dose, functional goals, and progression of rehabilitation targets are summarized in Table 1.

**Table 1:** Summary of the stage-based multidisciplinary rehabilitation program.

Intervention component	Dose and provider	Main goals and progression
Physical therapy/gross motor training	5 sessions/week, ∼40 min/session; pediatric physical therapists	Targeted head and trunk control, postural stability, transitional movements, lower-limb weight-bearing, balance, and gait preparation. Training progressed from prone support, rolling, and supported sitting to quadruped positioning, crawling, kneeling, supported standing, and gait-related practice.
Occupational therapy/fine motor and functional play	5 sessions/week, ∼40 min/session; occupational therapists	Focused on purposeful upper-limb use, hand–eye coordination, bimanual activity, functional play, and participation in daily routines. Tasks progressed from visual attention, reaching, and grasping to object transfer, bimanual play, simple manipulation, and imitation of daily activities.
Speech-language and early communication intervention	5 sessions/week, ∼30 min/session; speech-language therapists	Addressed early communication, social engagement, joint attention, imitation, turn-taking, and prelinguistic vocalization. Targets progressed from auditory/visual tracking and caregiver–child interaction to intentional communication and communication-promoting play.
Orofacial and feeding-related guidance	Integrated into speech-language therapy or caregiver training when indicated	Provided individualized support for oral-motor coordination, feeding efficiency, and feeding safety, including orofacial stimulation, feeding posture, pacing, and caregiver-mediated feeding strategies.
Orthotic management	Supramalleolar orthosis (SMO) introduced at approximately 20 months of age	Introduced before stable supported standing was achieved because of low muscle tone, insufficient ankle-foot stability, poor lower-extremity alignment, and the need to support standing training.
Caregiver-mediated home program	Caregiver-mediated activities were recommended for integration into daily routines as feasible.	Reinforced daily positioning, prone play, reaching, transitional movement practice, supported sitting/standing, upper-limb play, and communication-promoting interaction to support skill generalization.

Physical therapy focused on postural control, transitional movements, lower-limb weight-bearing, balance, and gait preparation, consistent with developmental motor intervention principles for children with early neuromotor impairment (5). Occupational therapy targeted purposeful upper-limb use, hand–eye coordination, bimanual activity, and functional play, in line with evidence supporting early intervention for children with developmental delay (6). Speech-language intervention emphasized early communication, social engagement, joint attention, imitation, turn-taking, and prelinguistic vocalization; oral-motor and feeding strategies were incorporated when clinically indicated (7). An SMO was introduced at approximately 20 months to support ankle-foot stability and standing training in the context of hypotonia (8). Caregivers were instructed to integrate positioning, motor practice, upper-limb play, and communication-promoting interaction into daily routines to support continuity and generalization of rehabilitation outside formal therapy sessions (9). Treatment plans were reviewed approximately every three months by the multidisciplinary team and adjusted according to standardized assessments, GMFM-88 domain scores, clinical observation, caregiver report, treatment tolerance, and participation. Center-based rehabilitation was scheduled Monday to Friday when the child was stable. Available records and caregiver reports suggested approximately 8 months of center-based rehabilitation per year, excluding holidays, short illness-related interruptions, and family absences. Overall tolerance was good, and no rehabilitation-related injury, therapy-associated other serious adverse event was documented.

### 2.4 Outcome measures

BSID-II was used to describe motor and mental development, including raw scores, developmental index values when available, percentile ranks, and age-equivalent estimates. Changes were interpreted as developmental progression rather than age-standardized treatment effects (10). GMFM-88 was used descriptively to track gross-motor skill acquisition across lying/rolling, sitting, crawling/kneeling, standing, and walking/running/jumping domains. The conventional total score was calculated as the mean of the percentages. As GMFM-88 has not been validated specifically for Dup15q syndrome, scores were interpreted as indicators of motor trajectory rather than disease-specific responsiveness (11). ADOS-2 was administered once at 2 years and 2 months by a trained evaluator to characterize autism-related social-communication and behavioral features; it was not treated as a longitudinal rehabilitation outcome (12).

### 2.5 Follow-up and outcomes

Longitudinal assessments and clinically relevant functional changes are summarized in Figure 2. To reduce redundancy, detailed values are presented in the figure, and the text highlights the main trajectory.

**Figure 2:**
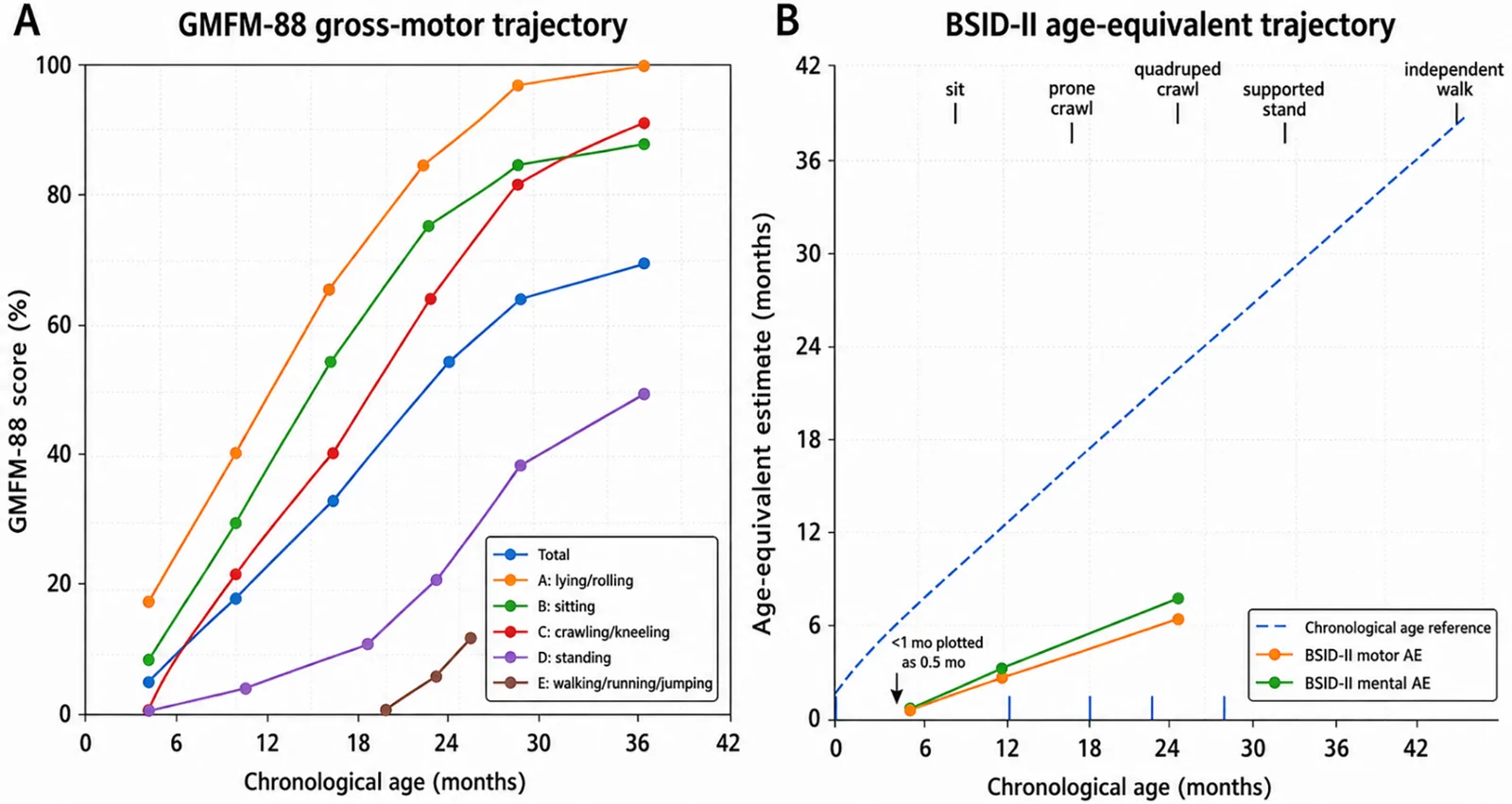
Longitudinal developmental and gross-motor trajectory. **(A)** GMFM-88 total and dimension percentage scores over time; the total score was calculated as the mean of the five dimension percentages. **(B)** BSID-II motor and mental age-equivalent estimates relative to chronological age; the dashed line indicates age equivalence. Milestones were based on retrospective therapist/caregiver reports.

BSID-II results showed gradual developmental progression, with the motor raw score increasing from 9 at 4 months and 28 days to 49 at 2 years and 1 month, and the mental raw score increasing from 19 to 71 over the same period, but persistent marked delay relative to chronological age. GMFM-88 results also demonstrated progressive gross motor gains, with the total score increased from 4.6% at baseline to 69.0% at 2 years and 11 months. Improvements were first observed in lying/rolling and sitting, followed by gains in crawling/kneeling, standing, and walking-related domains.

At 2 years and 2 months of age, ADOS-2 assessment yielded a total score of 21, including 20 points in the social affect domain and 1 point in the restricted and repetitive behavior domain, indicating clinically significant autism-related social communication impairment. Key functional milestones were incorporated into the main outcome summary to contextualize standardized scores. The child achieved stable head control at approximately 6 months, independent sitting at 12 months, prone crawling at 18 months, quadruped crawling at 21 months, supported standing at 24 months, and independent walking at 3 years and 4 months. He was also able to eat using an adaptive spoon, and eye contact was observed during interaction. These findings describe the child's developmental and functional trajectory during long-term multidisciplinary rehabilitation, rather than demonstrating treatment efficacy.

## 3 Discussion

This case describes the phenotype, rehabilitation management, and functional trajectory of a child with maternal Dup15q syndrome supported by a *de novo* maternally derived 15q11.2-q13.1 copy-number gain. The child presented with early feeding difficulty, delayed motor milestones, abnormal sleep EEG findings, delayed white matter maturation on MRI, and later autism-related social communication impairment. During follow-up, gradual gains were observed in developmental and gross motor measures, whereas social communication difficulties remained clinically significant. These findings should be interpreted as a descriptive developmental trajectory under multidisciplinary rehabilitation rather than as evidence of treatment efficacy.

The child's manifestations were consistent with the recognized maternal Dup15q phenotype, which includes developmental delay, hypotonia or motor impairment, language and communication deficits, seizures or abnormal EEG activity, intellectual disability, and ASD-related features (2, 13). Four copies across BP1-BP3 and approximately 75% SNRPN methylation supported maternally derived increased dosage. However, because karyotyping and FISH were not performed, the available data could not distinguish idic(15), interstitial triplication, or interstitial duplication. This distinction may affect severity, epilepsy risk, prognosis, and counseling, so we describe the finding as a maternally derived 15q11.2-q13.1 copy-number gain rather than assigning a structural subtype.

The BP1-BP3 interval includes neurodevelopmentally relevant genes. In addition to NIPA1, MAGEL2, and SNRPN, increased maternal dosage of UBE3A and the GABA receptor genes GABRB3, GABRA5, and GABRG3 may contribute to developmental, EEG/seizure, and autism-related phenotypes. These GABA receptor subunit genes are located within 15q11-q13 and have been investigated in ASD susceptibility because of their role in inhibitory neurotransmission, although a meta-analysis did not find a consistent association between common polymorphisms in these genes and ASD risk (14). These mechanisms remain speculative in this single case.

MRI findings included mildly enlarged extracerebral spaces at 4 months and reduced bilateral white-matter volume with abnormal periventricular signal at 11 months and 2 days, interpreted clinically as delayed white-matter maturation. These findings are not specific to Dup15q syndrome, and no later MRI was available. EEG showed occasional frontal sharp waves during sleep and abundant generalized fast activity during wakefulness and light sleep, but excessive fast beta activity was not explicitly quantified or labeled. The EEG abnormality and caregiver-reported seizure-like event nevertheless support continued neurological surveillance.

The rehabilitation program was early, staged, and multidisciplinary. Targets progressed from head and trunk control, prone support, rolling, and sitting to transitional movements, standing balance, and gait preparation. Physical therapy was combined with occupational therapy, speech-language intervention, feeding-related guidance, caregiver-mediated home activities, and later SMO support. The child's gains in BSID-II, GMFM-88, and milestones such as sitting, crawling, supported standing, and later walking indicate progressive functional acquisition during follow-up. However, without a comparator and given the rapid maturation of early childhood, the contributions of rehabilitation, natural development, family practice, and orthotic support cannot be separated.

Social-communication impairment remained significant despite motor progress. ADOS-2 findings were consistent with autism-related behavioral features reported in Dup15q syndrome (15). This dissociation between gross-motor gains and persistent social-communication difficulty suggests that phenotype-informed rehabilitation should set separate goals, monitoring strategies, and family expectations for motor and social-communication domains. Future studies may benefit from individualized measures such as Goal Attainment Scaling or the Canadian Occupational Performance Measure.

Several limitations should be noted. This was a retrospective single-case report based on available clinical records rather than a prospective protocol, so causality cannot be inferred. The structural subtype could not be resolved because karyotyping, FISH, and chromosomal microarray were unavailable. BSID-II was used because it was the authorized routine tool at the time, but this limits comparability with newer Bayley studies and floor effects were present. Early BSID-II gains may partly reflect improved test familiarity or baseline underestimation.

## 4 Conclusion

This case report documents the longitudinal developmental and functional trajectory of a child with maternal Dup15q syndrome supported by a *de novo* maternally derived 15q11.2–q13.1 copy-number gain undergoing early multidisciplinary rehabilitation. The observed gains in developmental and gross motor skills, together with persistent autism-related social communication impairment, emphasize the importance of individualized, phenotype-informed rehabilitation planning that addresses both motor function and social communication in children with rare neurodevelopmental disorders.

## Caregiver perspective

The child was too young to provide a direct personal perspective. His legal guardians reported that professional rehabilitation guidance improved their understanding of the child's developmental needs and provided practical strategies for positioning, play, communication, feeding, and mobility practice. They also described difficulty maintaining consistent home practice because of competing family-care responsibilities and the child's variable cooperation. They considered continued professional review and clear, feasible home recommendations important.

## Data Availability

The original contributions presented in the study are included in the article/Supplementary Material, further inquiries can be directed to the corresponding author.

## Supplementary material

Data Sheet 1 Completed CARE checklist for this case report.
