# Supplementary material for: Case Report: Early multidisciplinary rehabilitation and longitudinal functional outcomes in a child with a maternally derived 15q11.2-q13.1 copy-number gain
Source: Front Pediatr. 2026 Sep 14;14:1943381. doi: 10.3389/fped.2026.1943381 (PMC13617045; doi:10.3389/fped.2026.1943381)
Supplement: Supplementary file 1 [file Datasheet1.pdf]

CARE Checklist for Case Reports

Completed for the revised manuscript

Manuscript title: Case Report: Early Multidisciplinary Rehabilitation and Longitudinal Functional Outcomes in a Child with a Maternally Derived 15q11.2–q13.1 Copy-Number Gain

Authors: Jingyao Chen, Wangqingyuan Wang, Zijuan Wang, and Lihui Liao

Article type: Case Report

Checklist basis: CARE case report checklist items; locations are based on the current revised manuscript.

Note: Page and line numbers may change after final formatting or journal conversion. Section names and figure/table labels should be used as the primary location identifiers.

Item	Topic	CARE checklist item	Reported	Location in current manuscript
1	Title	The words "case report" appear in the title and identify the main phenomenon of interest.	Yes	Title page: "Case Report" and maternally derived 15q11.2–q13.1 copy-number gain.
2	Keywords	Key words identify diagnoses or interventions in this case report.	Yes	Keywords: Dup15q syndrome; maternally derived 15q11.2–q13.1 copy-number gain; pediatric occupational therapy; rehabilitation; developmental delay; case report.
3	Abstract	Concise summary of rationale, case presentation, interventions, outcomes, and main lesson.	Yes	Abstract: phenotype, genetic diagnosis, rehabilitation program, BSID-II/GMFM-88/ADOS-2 findings, milestones, and cautious interpretation.
4	Introduction	Brief background explaining why the case is unique, educational, or relevant.	Yes	Introduction: maternal Dup15q syndrome, phenotype heterogeneity, supportive management, and rationale for rehabilitation reporting.
5a	Patient information	De-identified demographic and clinical information.	Yes	Case description: male infant, age at referral, gestational age, birth weight/length, family history.
5b	Patient information	Chief concerns and symptoms.	Yes	Abstract/Case description: delayed milestones, feeding difficulty, early motor delay, reduced social responsiveness, hypotonia, later autism-related social-communication impairment.
5c	Patient information	Medical, family, and psychosocial history including relevant genetic information.	Yes	Patient information and Diagnostic assessment: non-consanguineous parents, negative family history, trio WES-CNV and MS-MLPA findings.
6	Clinical findings	Relevant physical examination and clinical findings.	Yes	Initial clinical findings: limited facial expression, reduced social responsiveness, narrow cranial contour, low-set ears, abnormal muscle tone, brisk lower-limb reflexes, unremarkable cardiopulmonary/abdominal examinations.
7	Timeline	Important dates and times in the case, preferably as a table or figure.	Yes	Figure 2: longitudinal developmental and gross-motor trajectory with GMFM-88/BSID-II results and milestones; Table 1: stage-based rehabilitation program.
8a	Diagnostic assessment	Diagnostic methods, including examination, testing, imaging, and genetic testing.	Yes	Diagnostic assessment: laboratory/metabolic testing, EEG, brain MRI, trio WES-CNV, MS-MLPA, karyotyping/FISH not performed.
8b	Diagnostic assessment	Diagnostic challenges, reasoning, differential diagnosis, and prognostic characteristics.	Yes	Diagnostic assessment/Discussion: interpretation of MRI/EEG, maternal-origin evidence, unresolved structural subtype, and paternal 1q22 VUS.
8c	Diagnostic assessment	Diagnosis including other possible diagnoses considered or excluded.	Yes	Diagnostic assessment: maternal Dup15q syndrome supported by de novo maternally derived 15q11.2–q13.1 copy-number gain; routine/metabolic screening did not identify an alternative diagnosis.
9a	Therapeutic intervention	Types of intervention and administration details.	Yes	Rehabilitation programs/Table 1: PT, OT, speech-language intervention, feeding-related guidance, caregiver-mediated home training, and SMO support.
9b	Therapeutic intervention	Dose, duration, frequency, and provider details.	Yes	Table 1: frequency/session duration/provider; Rehabilitation programs: approximately 8 months/year of center-based rehabilitation.
9c	Therapeutic intervention	Changes in intervention with explanations.	Yes	Rehabilitation programs/Table 1: stage-based progression; SMO introduced at approximately 20 months before stable supported standing.
10a	Follow-up and outcomes	Clinician- and patient-assessed outcomes when appropriate.	Yes	Follow-up and outcomes/Figure 2: BSID-II, GMFM-88, ADOS-2, and functional milestones.
10b	Follow-up and outcomes	Important follow-up diagnostic and intervention results.	Yes	Follow-up and outcomes/Discussion: developmental and gross-motor gains, persistent autism-related social-communication impairment, no later MRI, EEG surveillance context.
10c	Follow-up and outcomes	Intervention adherence and tolerability.	Yes	Rehabilitation programs/Caregiver perspective: schedule, estimated participation, interruptions/absences, good tolerance, no serious rehabilitation-related adverse events, variable home practice.
10d	Follow-up and outcomes	Adverse and unanticipated events.	Yes	Rehabilitation programs: no rehabilitation-related injury, therapy-associated seizure, or other serious adverse event was documented.
11a	Discussion	Strengths and limitations of the case report.	Yes	Discussion/Limitations: retrospective single case, no comparator, unresolved structural subtype, BSID-II limitations, floor effects, possible baseline underestimation.
11b	Discussion	Relevant medical literature.	Yes	Introduction/Discussion: Dup15q phenotype, rehabilitation literature, UBE3A/GABA receptor genes, ASD features, MRI/EEG interpretation, and Mahdavi et al. 2018.
11c	Discussion	Rationale for conclusions and clinical lessons.	Yes	Discussion/Conclusion: phenotype-informed rehabilitation, separate motor and social-communication goals, and cautious interpretation without causal inference.
12	Patient perspective	Patient or caregiver perspective when appropriate.	Yes	Caregiver perspective: legal guardians describe rehabilitation guidance, home-practice difficulty, and value of feasible recommendations.
13	Informed consent	The patient or legally authorized representative provided informed consent.	Yes	Ethics statement: written informed consent for publication of anonymized clinical information was obtained from the child's legal guardians.

Completed checklist prepared for submission with the revised case report manuscript.
